# The *Alternaria* genomes database: a comprehensive resource for a fungal genus comprised of saprophytes, plant pathogens, and allergenic species

**DOI:** 10.1186/s12864-015-1430-7

**Published:** 2015-03-25

**Authors:** Ha X Dang, Barry Pryor, Tobin Peever, Christopher B Lawrence

**Affiliations:** Department of Biological Sciences, Virginia Tech, Blacksburg, Virginia 24061 USA; Department of Plant Sciences, University of Arizona, Tucson, Arizona 85721 USA; Department of Plant Pathology, Washington State University, Pullman, Washington 99164 USA; Current address: Department of Internal Medicine, Division of Oncology, and The Genome Institute, Washington University School of Medicine, St. Louis, MO 63110 USA

**Keywords:** *Database*, *Alternaria*, *Fungal genome*, *Sequence*, *Annotation*, *Comparative genomics*, *Plant pathogen*, *Allergy*, *Saprophyte*

## Abstract

**Background:**

*Alternaria* is considered one of the most common saprophytic fungal genera on the planet. It is comprised of many species that exhibit a necrotrophic phytopathogenic lifestyle. Several species are clinically associated with allergic respiratory disorders although rarely found to cause invasive infections in humans. Finally, *Alternaria* spp. are among the most well known producers of diverse fungal secondary metabolites, especially toxins.

**Description:**

We have recently sequenced and annotated the genomes of 25 *Alternaria* spp. including but not limited to many necrotrophic plant pathogens such as *A. brassicicola* (a pathogen of Brassicaceous crops like cabbage and canola) and *A. solani* (a major pathogen of Solanaceous plants like potato and tomato), and several saprophytes that cause allergy in human such as *A. alternata* isolates. These genomes were annotated and compared. Multiple genetic differences were found in the context of plant and human pathogenicity, notably the pro-inflammatory potential of *A. alternata*. The *Alternaria* genomes database was built to provide a public platform to access the whole genome sequences, genome annotations, and comparative genomics data of these species. Genome annotation and comparison were performed using a pipeline that integrated multiple computational and comparative genomics tools. *Alternaria* genome sequences together with their annotation and comparison data were ported to Ensembl database schemas using a self-developed tool (EnsImport). Collectively, data are currently hosted using a customized installation of the Ensembl genome browser platform.

**Conclusion:**

Recent efforts in fungal genome sequencing have facilitated the studies of the molecular basis of fungal pathogenicity as a whole system. The *Alternaria* genomes database provides a comprehensive resource of genomics and comparative data of an important saprophytic and plant/human pathogenic fungal genus*.* The database will be updated regularly with new genomes when they become available. The *Alternaria* genomes database is freely available for non-profit use at http://alternaria.vbi.vt.edu.

## Background

*Alternaria* species are a major cause of necrotrophic diseases of plants and some of the most common fungi encountered by humans. There are several noteworthy examples of *Alternaria* spp. as major plant pathogens including but not limited to, *A. brassicicola* and *A. solani. A. brassicicola* causes black spot disease (also called dark leaf spot) on virtually every important cultivated *Brassica* spp. [1-3]. Black spot disease is of worldwide economic importance. For example, black spot can be a devastating foliar and seed-borne disease resulting in severe yield reductions in crops such as cabbage, broccoli, canola and rapeseed [4-6]. *A. solani* is the causal agent of early blight disease of several major *Solanaceous* crops including potato and tomato. Early blight caused by *A. solani* is considered one of the most destructive diseases of potatoes and tomatoes in the world [7,8].

*Alternaria spp.* are among the most well known producers of diverse secondary metabolites, especially toxins [9]. Over 70 small molecule compounds have been reported from *Alternaria* [9]. Some of these metabolites are potent mycotoxins (e.g. alternariol, alternariol methyl ether, tenuazonic acid, etc.) with mutagenic and teratagenic properties, and have been linked to certain forms of cancer [10]. The occurrence of potentially harmful *Alternaria* metabolites in food and food products is becoming an increasing environmental concern [11]. Other toxins are host specific or non-host specific phytotoxins and are important virulence factors during plant pathogenesis. To date many of the genes responsible for the production of these specialized metabolites are unknown although recently the genes responsible for production of the HDAC inhibitor depudecin in *A. brassicicola* was elucidated as well as the toxin Alternariol and Alternariol methyl ether in *A. alternata* [12-14]. Annotated genome sequence information was critical for these discoveries.

In addition to harboring many important plant pathogenic species, *Alternaria* spores are one of the most common and potent indoor and outdoor sources of airborne allergens. Epidemiological studies from a variety of locations worldwide indicate that *Alternaria* sensitivity is closely linked with the development of atopic asthma and up to 70% of mold-allergic patients have skin test reactivity to *Alternaria* [15-17]. *Alternaria* sensitivity has been shown to not only be a risk factor for asthma, but can also directly lead to the development of severe and potentially fatal asthma often more than any other fungus [15-19]. Although some research has been performed on the physiological and molecular identification of *Alternaria* allergens only three major and several minor allergenic proteins have been described [20]. The biological role of these allergens and other secreted fungal products in the development of allergy and asthma is very poorly understood. Thus there is clearly a need to elucidate the role of *Alternaria* immunoreactive proteins and other molecules such as secondary/specialized metabolites in the development of allergic diseases from both diagnostic and immunotherapeutic perspectives.

In this article, we introduce the *Alternaria* genomes database that provides tools to browse and visualize genome sequences, genome annotations, whole genome alignments, and homologous data of the fungal genus *Alternaria*.

## Content and construction

The *Alternaria* genomes database houses genome sequences, genome annotation and genome comparison data from 25 species, including saprophytes, necrotrophic plant pathogens and species associated with human diseases like allergic airway disorders (Table 1). These genomes were analyzed using a pipeline that incorporated multiple computational and comparative genomics tools. Genomes (i.e. genomic sequences, in the form of contigs or supercontigs) were assembled from Sanger or next-generation sequencing reads and then used as the input for the pipeline. These sequences were analyzed through multiple annotation modules, including repetitive sequence annotation, gene prediction, protein function and domain structure annotation. Comparative genomics analyses were also performed including whole genome alignment and homology analysis.

**Table 1:** **Description of the sequenced** ***Alternaria*** **genomes**

**Species name**	**Strain codes**	**Additional information**	**Sequencing technologies**	**Genome sequence size (Mb)**	**Contigs/super-contigs**	**Contigs/super-contigs N50(kb)**	**Predicted genes (#)**
*A. alternata*	ATCC 66891, EGS 34–016, BMP 0269	Allergic diseases of human, leaf spot, rots of plants	454	33.2	499	300	11635
*A. alternata*	ATCC 11680, BMP 0238, IHEM 4706	Allergic diseases of human, leaf spot, rots of plants (possibly *A. tenuissima*)	454	33.8	797	450	12323
*A. brassicicola*	ATCC 96836, EGS 42–002, BMP 1950	Blackspot of brassica	Sanger	29.6	4039/838	18/ 2400	10514
*A. alternata*	ATCC 66982, EGS 34–039, BMP 0270	Allergic disease of human, leaf spot, rots of plants	Illumina	33.5	393	757	12290
*A. arborescens*	ATCC 204491, EGS 39–128, BMP 0308	Stem canker of tomato	Illumina	34.0	1332	624	14741
*A. citriarbusti*	EGS 46–140, BMP 2343, SH-MIL-8 s	Brown/black spot of citrus	Illumina	34.1	2273	48	12606
*A. destruens*	ATCC 204363, EGS 46–069, BMP 0317	Infecting and suppressing dodder (weed)	Illumina	41.8	31070	3	14814
*A. fragariae*	BMP 3062, NAF-8	Black spot disease of strawberry	Illumina	33.2	1027	78	12272
*A. gaisen*	EGS 90–0512, BMP 2338	Black spot, ring spot disease of pear	Illumina	34.6	7485	10	13902
*A. tangelonis*	EGS 45–080, BMP 2327, BC2-RLR-1 s	Leaf spot of citrus	Illumina	34.0	2459	37	12639
*A. longipes*	EGS 30–033, BMP 0313	Black/brown leaf spot of tobacco	Illumina	36.3	3412	137	13219
*A. mali*	BMP 3064, IFO8984	Leaf ring spot of apple	Illumina	34.7	2682	35	12715
*A. mali*	BMP 3063, M-71	Leaf ring spot of apple	Illumina	34.1	4439	21	12727
*A. turkisafria*	BMP 3436, SH-MIL-20s	Leaf spot of citrus	Illumina	34.0	2347	33	12739
*A. tenuissima*	ATCC 96828, EGS 34–015, BMP 0304	Leaf spot of plants	Illumina	33.5	676	662	12276
*A. limoniasperae*	EGS 44–159, BMP 2335	Leaf spot of citrus	Illumina	35.1	2796	50	12966
*A. carthami*	BMP 1963, CBS 635.80	Leaf spot and blight of safflower	Illumina	34.5	9340	72	12071
*A. capsici*	ATCC MYA-998, EGS 45–075, BMP 0180	Leaf spot of solanaceae (pepper)	Illumina	34.0	13743	31	11487
*A. crassa*	BMP 0172, ACR1	Leaf spot of solanaceae	Illumina	35.0	12126	54	11663
*A. dauci*	ATCC 36613, BMP 0167	Leaf blight of carrots	Illumina	32.1	12030	13	11981
*A. macrospora*	BMP 1949, CH3	Leaf spot of cotton	Illumina	31.7	3153	37	11961
*A. porri*	BMP 0178, Z6B	Purple blotch, leaf blight and bulb rot of Allium (onion)	Illumina	31.2	16767	9	12232
*A. solani*	BMP 0185	Early blight of potatoes and tomatoes	Illumina	32.9	5613	144	11726
*A. tagetica*	EGS 44–044, BMP 0179	Leaf spot of marigold	Illumina	35.1	16372	72	11999
*A. tomatophila*	BMP 2032, CBS 109156	Leaf spot of tomato	Illumina	34.1	10185	22	12601

### Genome sequencing and assembly

*Alternaria* genomes were sequenced using various sequencing technologies including whole genome shotgun method with Sanger sequencing, GS-FLX 454, and Illumina HiSeq (Table 1). Genomes were assembled from sequencing reads using PCAP [21] (for Sanger sequencing), Newbler [22] (for GS-FLX 454), and Velvet [23] (for Illumina HiSeq). The physical map of *A. brassicicola* was constructed by generating fingerprints from the CSU-K35 *A. brassicicola* BAC library that were then used to scaffold the genome (Dang et al., unpublished).

### Genome annotation

**Figure 1:**
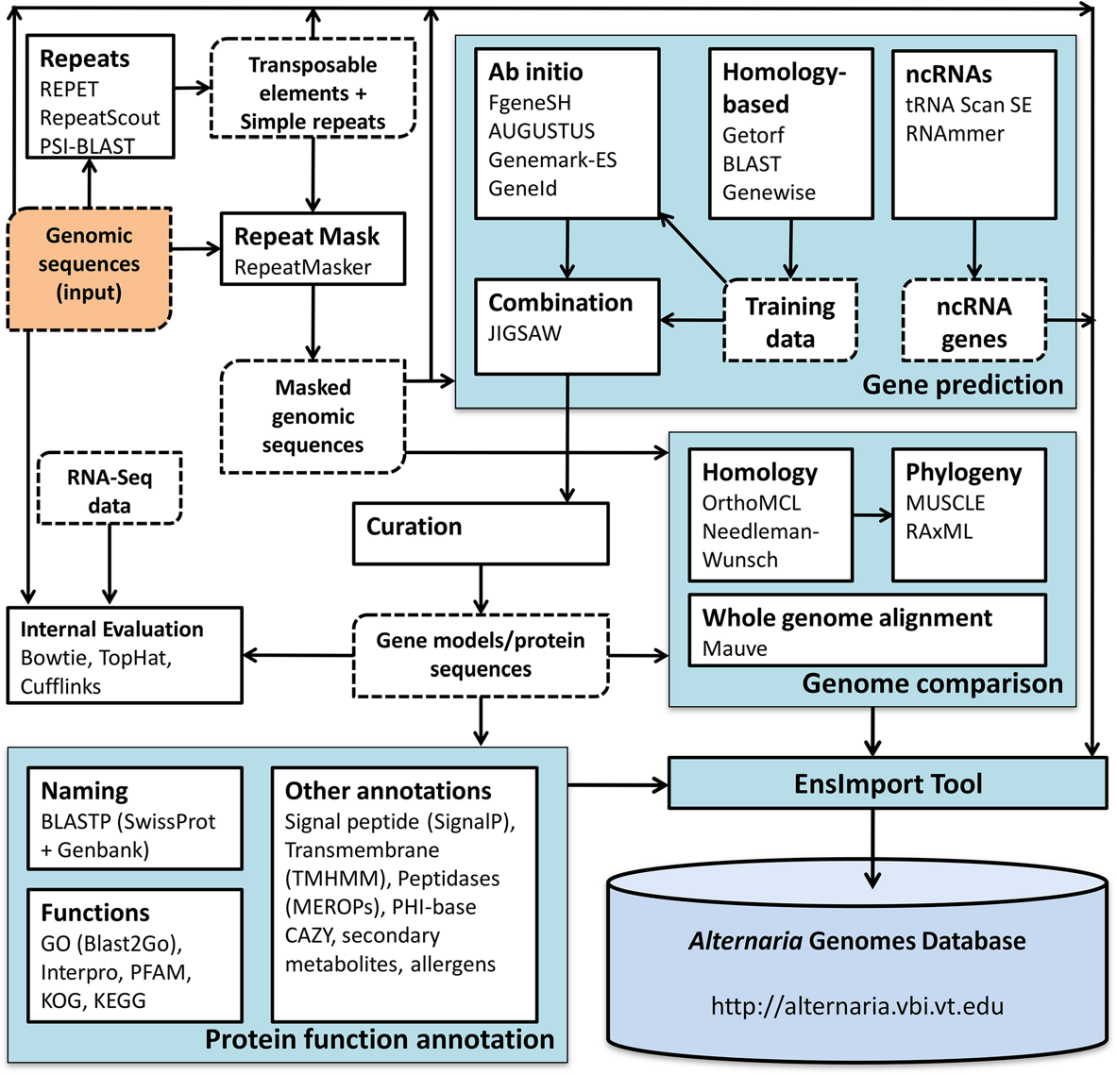
***Alternaria*** **genome annotation pipeline.**

Genome annotation was performed using a custom pipeline (Figure 1). Assembled genomes were first scanned for repetitive sequences (both transposable elements and simple repeats) using multiple tools including REPET [24], RepeatScout [25], RepeatModeler and RepeatMasker (http://www.repeatmasker.org). Protein-coding gene prediction was then carried out using JIGSAW [26] that combined gene models discovered by various de novo and homology-based gene prediction tools including Genewise [27], FgeneSH (http://softberry.com), AUGUSTUS [28], Genemark-ES [29], and GeneID [30]. We also generated RNA-Seq data for *A. alternata ATCC 66981* which were aligned to the genome using TopHat [31] with Bowtie [32], and de novo transcripts were constructed using Cufflinks [33]. These data were used internally to evaluate gene predictions. Predicted genes were then conceptually translated to protein sequences that served as the input for most of the functional annotation tasks. Non-coding genes were also annotated using tRNAScan-SE [34] and RNAmmer [35].

Various computational functional annotations were performed on the conceptual protein sequences. The proteins were first searched against Genbank [36] and SwissProt [37] using BLAST to identify known proteins with similar sequences. The name/description of the known proteins was then transferred to the predicted proteins following the standard operating procedure (SOP) developed for fungi by the Broad Institute [38]. Protein domain and family annotation was performed using the Interpro database [39] and PFAM [40]. Gene ontology annotation was performed using Blast2GO [41] and Interpro.

Various fungal-related and additional annotations were also carried out using the pipeline. Signal peptides were predicted using SignalP [42], WoLF-Psort [43], and Phobius [44]. Transmembrane proteins were predicted using TMHMM [45]. Pathogenicity-related gene candidates were identified via multiple annotation data including BLAST search against PHI-base [46]. Carbohydrate Active Enzymes were identified according to the CAZY database [47] and dbCAN [48]. Potential allergens were identified using BLAST based homology searches and Allerdictor [49]. Proteases were annotated using the batched BLAST search tool from the MEROPS database [50]. Secondary metabolites were identified using SMURF [51].

### Genome comparison

Multiple genome comparison tasks were performed that utilized the genome sequences as well as the predicted genes/proteins from multiple species. Whole genome pairwise alignment was performed using Mauve progressive alignment software [52,53]. Orthologs and paralogs were identified using bidirectional best BLAST hits and Markov clustering via OrthoMCL [54].

### Porting data to Ensembl database schema

Annotation and comparison data of *Alternaria* genomes are presented via the popular Ensembl genome browser platform [55] that was customized and installed at the Virginia Bioinformatics Institute. Outputs from the genome annotation pipeline as well as outputs from comparative genomics analyses were processed and converted to Ensembl compatible MySQL databases (both core and compara databases) using EnsImport, a custom suite of scripts we developed in Perl. EnsImport supports multiple standard file formats such as FASTA, AGP, GFF3 and XMFA, and outputs from widely-used tools such as BLAST, Interpro, RepeatMasker, OrthoMCL and Blast2GO.

## Utility and discussion

Using Ensembl genome browser platform, the *Alternaria* genomes database provides a rich set of user-friendly tools to browse and visualize sequences, annotation, and comparison data. Data export and search features are also available. Detailed instructions on how to use the Ensembl browser are available on the ‘Help & Documentation’ section of the database. Here we only describe the most relevant features in the context of the *Alternaria* genomes project.

### Genome region view

**Figure 2:**
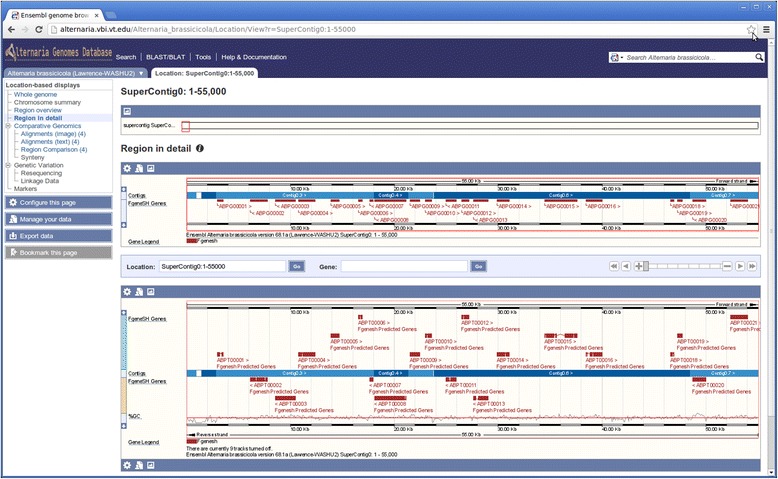
**A screenshot of the** ***Alternaria*** **genomes database that shows a region of an** ***A. brassicicola*** **supercontig along with the predicted genes and transcripts.**

For each species, users can access and visualize a genomic region along with annotated functional and non-functional elements such as repetitive elements, predicted protein-coding gene models, and RNA coding gene models (Figure 2). A genomic region can be a whole (or part of) a contig or supercontig. Zooming functionality allows for intuitively scaling region views based on location. Each type of element (functional and non-functional) is displayed in a separate track using a unique color. Users can click on an individual element (e.g. repeats, genes, transcripts) to open a popup menu to access available annotation. The tracks can be displayed or hidden using the display configuration tool.

### Annotation view

**Figure 3:**
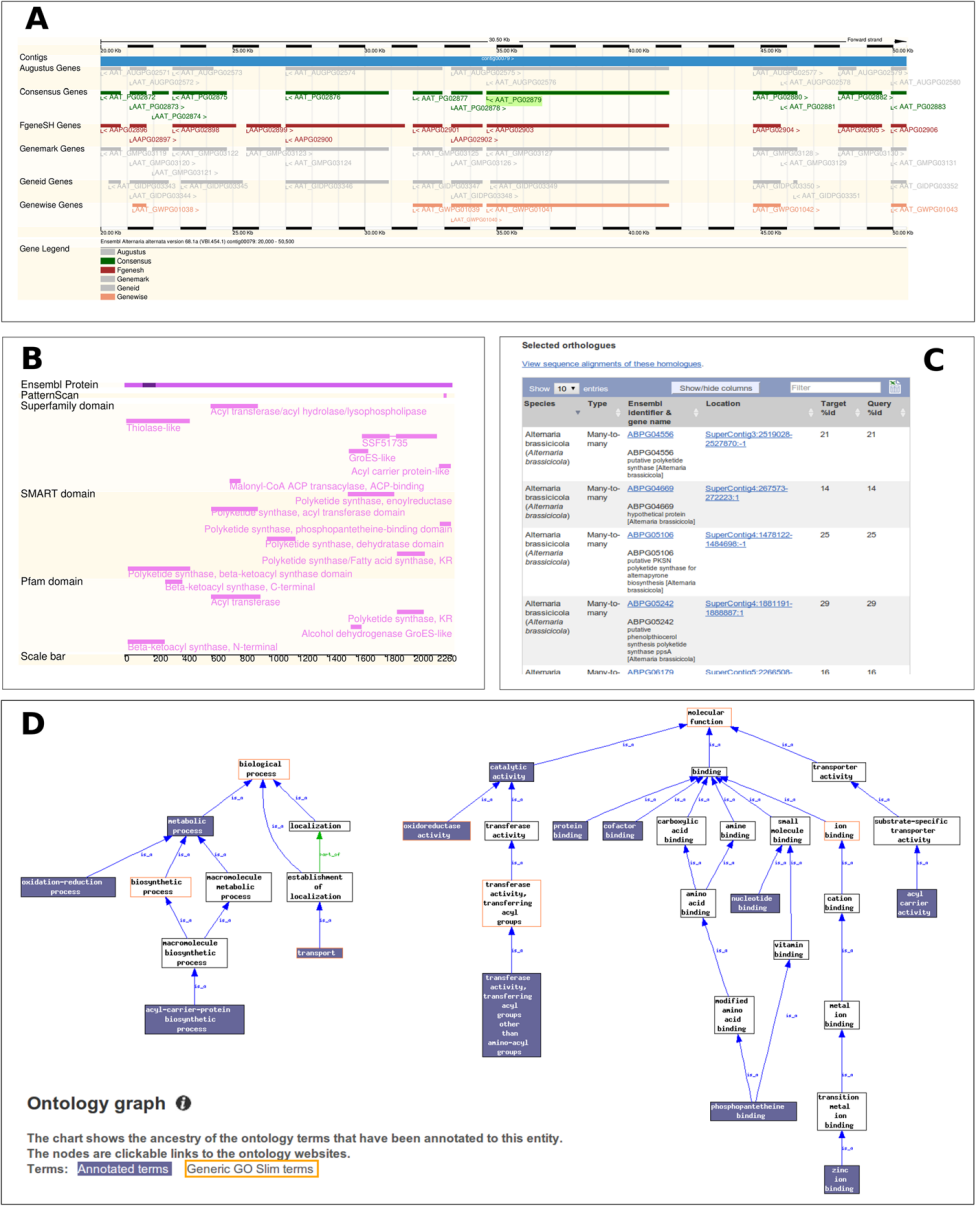
**Examples of annotation and comparison views for an** ***Alternaria alternata*** **polyketide synthase gene (AAT_PG02879). (A)** Contig view of the gene, **(B)** Domain annotation, **(C)** Orthologous genes in other *Alternaria* genomes, **(D)** Gene ontology annotation.

The majority of functional annotation data in the database is for protein coding genes. For each gene/protein, extensive annotations include gene structure and sequence, gene description, location, protein domain architectures (e.g. Interpro, PFAM), gene ontology assignments, signal peptides, transmembrane structures and other annotation data (Figure 3). These annotation data are available and presented in multiple tightly linked web interfaces in the browser.

### Comparative genomics view

**Figure 4:**
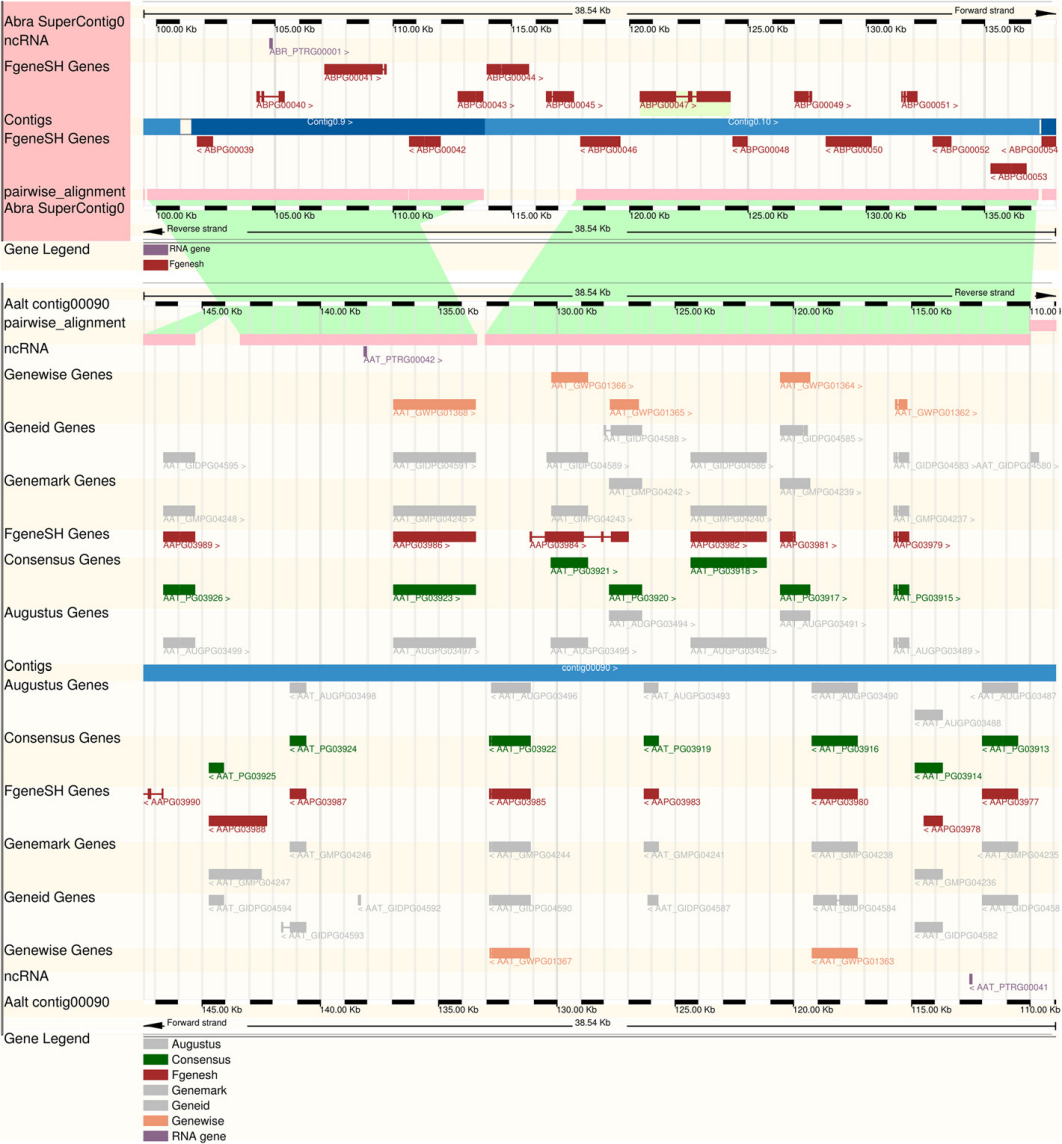
**An example of a syntenic region between** ***Alternaria brassicicola*** **and** ***A. alternata*** **. The aligned blocks (in pink) between genomic sequences are connected by green bands.**

The comparative browsing feature of Ensembl platform allows for conveniently viewing and visualizing comparative genomics data side-by-side with annotation data. Aligned regions between two genomes identified via whole genome pairwise alignments are displayed together with functional and non-functional elements such as repetitive elements and gene models (Figure 4). This feature allows for easy investigation of the conserved genomic regions between multiple genomes. Whole genome alignments can be visualized using graphical representation as well as displayed in text formats such as FASTA and ClustalW. Orthologs and paralogs of a gene can be easily retrieved in a table that contains links to access protein alignments and related annotation data (Figure 3C).

### Database search

**Figure 5:**
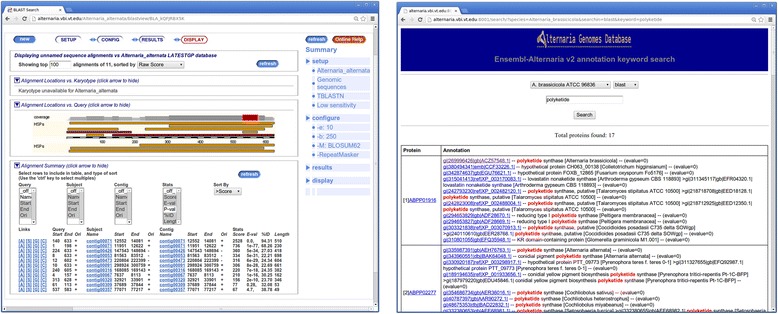
**Search features of** ***Alternaria*** **genomes database that allows for sequence alignment search using BLAST (left) and Interepro and BLAST hit description search (right).**

Users may query the database using sequence alignment search (e.g. BLAST) and text search. The built-in search feature of the Ensembl platform allows for BLAST searches against genomic sequences, predicted transcript and protein sequences (Figure 5). Full text search for gene names is also available as a built-in feature in Esembl platform. However, for newly sequenced species, a large portion of the predicted genes are not named or annotated with highly reliable descriptions. In such cases, information on the hits with known proteins or protein families and domains can be used to explore the functions of the genes. Therefore, we implemented a more comprehensive search module that allows for full text search within annotation from multiple sources including BLAST and Interpro hits and incorporated this module in the *Alternaria* genomes database (Figure 5).

### Data export

Ensembl built-in functionality allows for exporting multiple types of data to various formats. Raw sequence and annotation data can be easily exported in multiple formats such as FASTA and GFF via available tools in Ensembl. A button to access data export feature is located on the left pane in the interface of the database. It is also possible to export the graphical visualization of multiple types of annotation and comparison data to multiple image formats that are suitable for publication or further editing.

## Conclusion

Over the past few years, efforts in sequencing fungal genomes have facilitated the studies of the molecular basis of fungal pathogenicity as a whole system [56-59]. The *Alternaria* genomes database provides a comprehensive resource of genomics and comparative genomics data of an important plant and human pathogenic fungal genus *Alternaria.* In addition, the database may prove useful for discovery of genes encoding industrial enzymes, antibiotics, and other molecules with utility in medicine and agriculture.

These genome annotation and comparison data have recently facilitated several large-scale functional genomics studies that resulted in the discovery of many new genes that contribute to virulence especially secondary metabolite genes, mitogen-activated protein (MAP) kinases, and transcription factors in *A. brassicicola* [13,14,60-68]. *Alternaria* genome annotation and comparison data have also enabled comprehensive comparative studies of *Alternaria* genomes in the context of plant and human pathogenicity [69] (several other manuscripts are under preparation).

The use of the familiar Ensembl browser platform makes browsing and visualizing *Alternaria* genome annotation and comparison data convenient. As we continue our efforts in *Alternaria* genome sequencing and analysis, we will update this database as new genomes and relevant annotation data become available.

## Availability and requirements

The *Alternaria* genomes database is freely available for non-commercial use at http://alternaria.vbi.vt.edu.
